# A bibliometric and visualization analysis of global trends and frontiers on macrophages in abdominal aortic aneurysms research

**DOI:** 10.1097/MD.0000000000040274

**Published:** 2024-10-25

**Authors:** Liang Zhang, Dongyu Li, Shiyang Bao

**Affiliations:** a Department of Breast Surgery, Hubei Provincial Clinical Research Center for Breast Cancer, Wuhan Clinical Research Center for Breast Cancer, Hubei Cancer Hospital, Tongji Medical College, Huazhong University of Science and Technology, Wuhan, Hubei, China; b Department of VIP In-Patient Ward, The First Hospital of China Medical University, Shenyang, Liaoning, China.

**Keywords:** abdominal aortic aneurysms, bibliometric analysis, citespace, immune-microenvironment, macrophage, mechanism research

## Abstract

**Background:**

Macrophages are key regulators of the inflammatory and innate immune responses. Researchers have shown that aberrant expression of macrophages contributes to the development of abdominal aortic aneurysms (AAA). However, a comprehensive bibliometric analysis exploring the research status and knowledge mapping of this area is lacking. This study aimed to explore the research status, knowledge mapping and hotspots of macrophages in AAA research from a bibliometric perspective.

**Methods:**

In this study, we retrieved articles published between 2000 and 2022 on macrophages associated with AAA research from the Web of Science Core Collection (WoSCC) database. The retrieved literature data were further analyzed using Citespace and VOSviewer software.

**Results:**

A total of 918 qualified publications related to AAA-associated macrophages were retrieved. The number of publications in this field has been increasing annually. China and the United States were the 2 main drivers in this field, contributing to more than 64% of the publications. In addition, the US had the most publications, top institutions, and expert researchers, dominating in research on macrophages in AAA. The Harvard University was the most productive institution, with 60 publications. The journal with the most publications was Arteriosclerosis, Thrombosis, and Vascular Biology (86). Daugherty Alan was the most prolific author (28 publications) and he was also the most cited co- author. Furthermore, the exploration of established animal models, macrophage-related inflammatory-microenvironment, macrophage-related immune mechanism, clinical translation and molecular imaging research remained future research directions in this field.

**Conclusions:**

Our findings offered new insights for scholars in this field. They will help researchers explore new directions for their work.

## 1. Introduction

Cardiovascular disease is a chronic, complex disease of the heart and circulatory system and has high rates of illness and death.^[1]^ Abdominal aortic aneurysms (AAA), one of the most potentially life-threatening cardiovascular diseases, is a chronic life-threatening disease which is characterized by abnormal dilation of the abdominal aorta. AAA occurs when the abdominal aorta pathologically dilates to over 1.5 times its normal diameter. AAA is extremely dangerous because it has no symptoms in the early stages. Current clinical treatment for AAA is limited to surgery.^[2]^ For small aneurysms (3.0–5.5 cm), no effective drugs can prevent their progression. Thus, knowledge of the molecular mechanism behind AAA is essential.^[3]^

Inflammatory cell infiltration, extracellular matrix (ECM) remodeling and loss of vascular smooth muscle cells (VSMCs) are central part in AAA development.^[4]^ Numerous studies suggested that effective drugs against vascular inflammation could inhibit AAA progression.^[5–7]^ Macrophages regulate inflammatory and immune responses and have been proven to play a central role in the pathogenesis of AAA. In the media and adventitia layer of AAA samples, macrophages are the predominant cell type.^[8]^ Under pathological conditions, early monocytes infiltrate into the aortic media and adventitia and further differentiate into the inflammatory macrophage subset, contributing to the destruction of the aortic wall. Macrophage polarization has been well-studied in AAA model. It’s reported that blockage of macrophage migration is effective in inhibiting AAA progression. A recent study by Dang et al^[9]^ revealed that targeting the T lymphocyte-extracellular vesicles-macrophage axis inhibited AAA progression. Hans et al^[10]^ found that decreased level of Notch1 caused defects in macrophage migration and proliferation, further attenuating AAA progression.

Researchers have studied macrophages in AAA for many years, however, no bibliometric studies in this field exist. This study retrieved relevant bibliometric data, including annual publications, countries, authors, institutions, keywords and references. This study systematically illustrated the research trend and frontiers of macrophage related to AAA from 2000 to 2022. It provides valuable data for further study.

## 2. Methods

### 2.1. Data collection and search strategy

**Figure 1.**
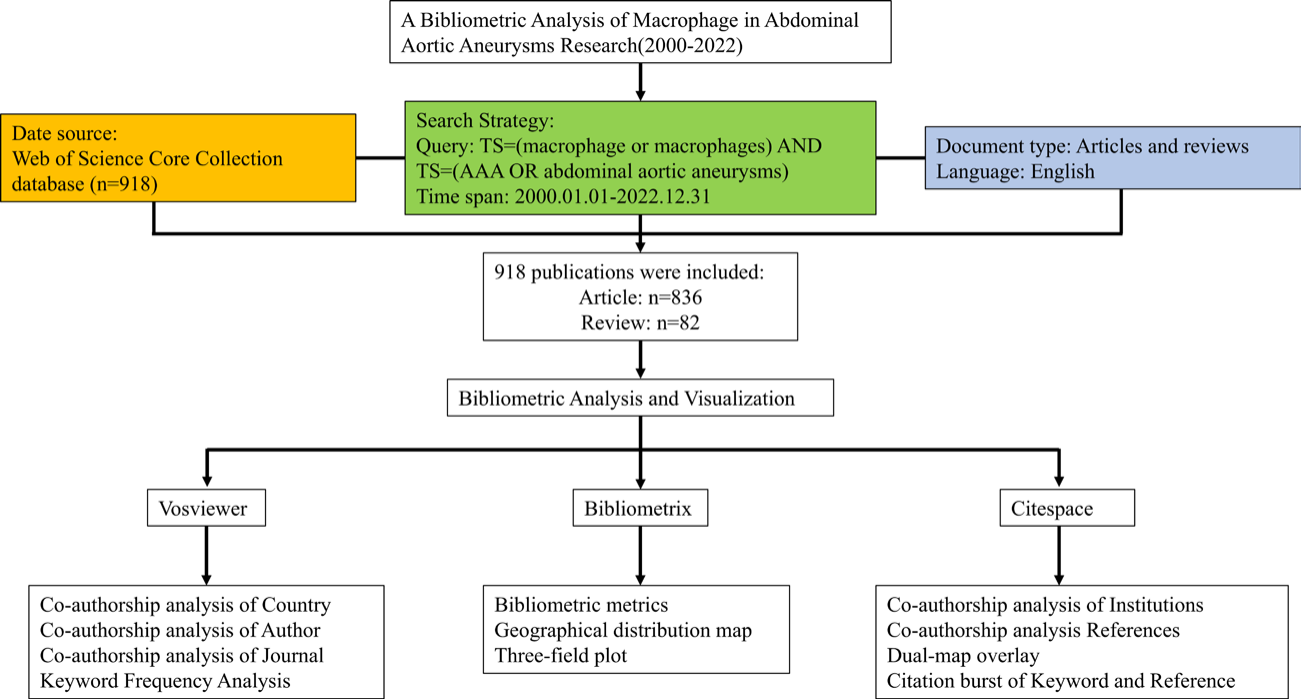
Workflow for choosing publications in the present study.

The whole study was conducted in accordance with the Declaration of Helsinki. The scope of this study excluded animal and human experimentation, so there are no relevant ethical issues involved in this study. The Web of Science Core Collection (WoSCC) database contained a huge amount of publications and was the best database for bibliometric analysis.^[11]^ This study extracted global published articles from the WoSCC database (editions: Science Citation Index Expanded [SCI-Expanded] and Social Sciences Citation Index). To ensure the accuracy, the data was collected by 2 independent authors (Shiyang Bao and Dongyu Li) in 1 day (October 17, 2023). The retrieval strategy was TS = (macrophage or macrophages) and TS = (AAA or abdominal aortic aneurysms). The time span of this study was set as January 01, 2000 to December 31, 2022. This study retrieved articles and reviews, and limited the language to English. Furthermore, all the coauthors identified any possible discrepancies and obtained 918 publications for further analysis. The detailed search strategy of this study was shown in Figure 1.

### 2.2. Data collection and bibliometric analysis

**Figure 2.**
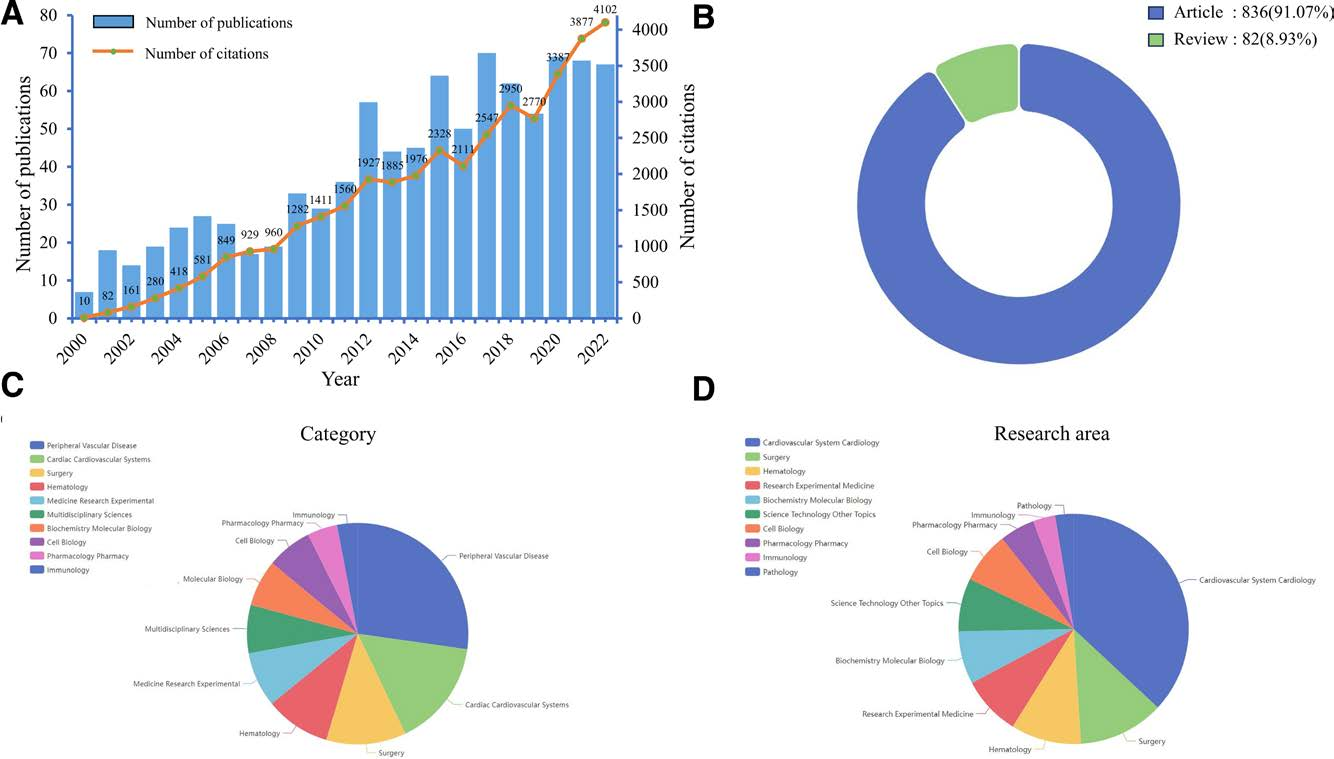
Overview of macrophages in AAA research from 2000 to 2022. (A) The annual trend of publications and citations from 2000 to 2022. (B) Distribution of publications by type. (C) The top 10 research categories. (D) The top 10 research areas. AAA = abdominal aortic aneurysm.

The raw data was retrieved from WoSCC database and saved in plain text format. Then, the acquired data was imported into the Citespace 6.4.R1 software (a JAVA-based data visualization software designed by Dr Chen Chaomei) for bibliometric analysis. This software reveals trends in knowledge domains over specific time periods. The parameters of Citespace were set as follows: time span (2000–2022), years per slice (1). In this study, we used Citespace software to analyze core institution, keywords and references. We also examined trends in keyword and reference bursts. In addition to Citespace, we utilized Vosviewer, another powerful literature processing tool, for further analysis. We used Vosviewer software to analyze country collaboration, authors, active journals and keywords in detail. GraphPad Prism 6 was used to plot annual publication output. Then, we used R package “bibliometrix” to visualize annual country’s publication and map international collaboration among countries. Apache ECharts tool (https://echarts.apache.org/en/index.html), an open-sourced JavaScript visualization tool, was used to create a highly- customizable pie chart (Fig. 2). Also, we obtained the H-index of scholars, the impact factor (IF) and journal citation reports (JCR) division of journals from the Web of Science (WOS) database.

## 3. Results

### 3.1. General trends and annual publications

The workflow of this study is shown in Figure 1. After removing invalid publications, a total of 918 publications from WoSCC database were retrieved from 2000 to 2022, including 836 articles and 82 reviews (Fig. 2B). The histogram in Figure 2A has shown that the annual publications related to macrophages in AAA research from 2000 to 2022 have increased rapidly year by year. The category and research area of the retrieved publications are shown in Figure 2C,D. Our results have shown that the growth trend of the annual publications and citations in this area has been increasing steadily in the past 22 years. The annual number of publications reached the bottom in 2000 (7) and peaked in 2017 (70). This number has increased almost 10-fold in the past 20 years. The annual number of citations grew steadily as well. We divided into 2 periods: the slow growth period (2000–2008) and the rapid growth period (2009–2022). The number of publications increased slowly before 2008 and rapidly after 2009 and reached a peak in 2017 (70). Taken together, these findings indicated that the research of macrophage in AAA research has gained increasing attention and reached a stage of rapid development in the past 20 years and is likely to increase in the future.

### 3.2. Distribution of countries/regions

The total number of publications over a certain period reflected the field’s research trend quantitatively. From 2000 to 2022, a total of 918 publications related to macrophages in AAA research published by 34 countries were retrieved. 24 countries contributed to more than 5 publications. The top 10 most productive countries were listed in Table 1. The USA had the largest number of publications (377), followed by China (210), Japan (171), Germany (67), the United Kingdom (51), France (46), Sweden (31), Netherlands (31) Australia (28) and Spain (24).

**Table 1:** The top 10 countries with the most publications.

Rank	Country/region	Publications	Total citations	Average citations	Percentage (%)
1	USA	377	23,514	62.37135	41.07
2	China	210	4462	21.24762	22.88
3	Japan	171	5634	32.94737	18.63
4	Germany	67	3156	47.10448	7.30
5	The United Kingdom	51	2837	55.62745	5.56
6	France	46	1915	41.63043	5.01
7	Sweden	31	1826	58.90323	3.38
8	Netherlands	31	1636	52.77419	3.38
9	Australia	28	915	32.67857	3.05
10	Spain	24	531	22.125	2.61

**Figure 3.**
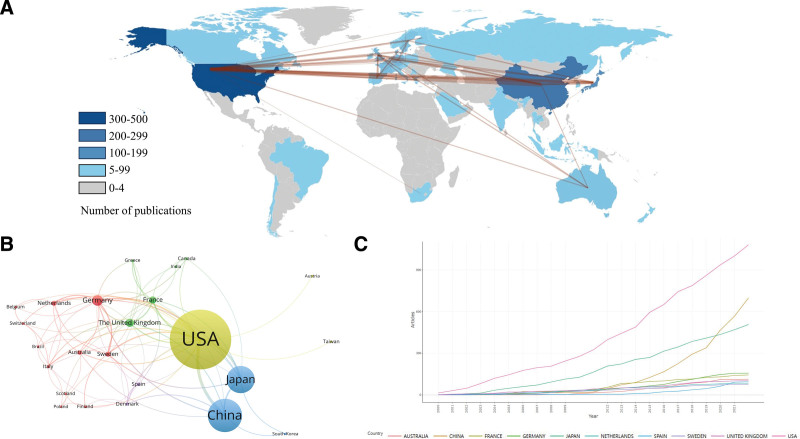
Network visualization map of countries involved in macrophages on AAA research. (A) Geographical distribution map of global publications in the field of macrophages in AAA research. The number of publications the thickness of lines among countries represents their cooperative strength/frequency. (B) Analysis of cooperation among countries based on VOSviewer software. In the networks, the node size represents the number of publications. The larger the size is, the more publications the country has made to this field. The lines between nodes represents the collaboration of the item. The thicker the line represents the closer the relationship among countries. Each node represents a different country, the larger the node, the more publications; the wider the link between the nodes, the closer the collaboration between the countries. (C) Annual growth trends of the top 10 most productive countries from 2000 to 2022. AAA = abdominal aortic aneurysm.

The total number of publications from the top 3 countries accounted for more than 80% of the total publications (Fig. 3A). The top 3 countries with the highest average citations were the USA, Sweden and the United Kingdom, indicating results from these countries were relatively mature. To discover potential collaboration among countries, the collaboration network was generated by using the VOSviewer software. As shown in Figure 3B, extensive collaboration among countries was observed. The USA and China were the major collaborators in this field. Figure 3C depicted the top 10 most productive countries’ annual publications from 2000 to 2022. The USA was still the most productive and active country, followed by Japan and China. But since 2020, China published more studies than Japan and became the second productive country in this field.

### 3.3. Distribution of institutions and analysis of authors

**Figure 4.**
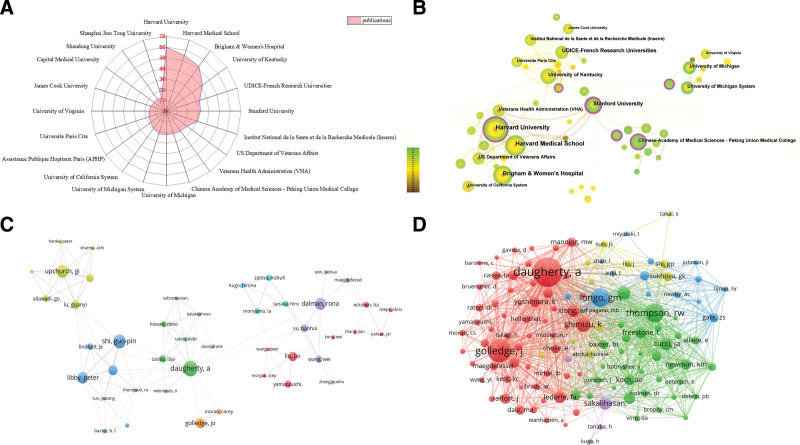
Network visualization map of institutions and authors involved in macrophages on AAA research. (A) Radar chart showing the top 10 most productive institutions involved in macrophages in relation to AAA. (B) Analysis of cooperation among institutions based on Citespace software. (C) Visualization map of 51 authors with >7 publications (7 of the 51 authors are not connected to each other). The line between nodes reflects cooperation between authors. (D) Visualization map of co-citation analysis among authors. AAA = abdominal aortic aneurysm.

In the present study, a total of 5071 authors from 1091 institutions had made contributions in this field. As shown in Table 2, the most productive institution was Harvard University (60), followed by Harvard Medical School (55), Brigham & Women’s Hospital (52), University of Kentucky (42), UDICE-French Research Universities (33), Stanford University (32), Institut National de la Sante et de la Recherche Medicale (Inserm; 31), US Department of Veterans Affairs (24), Veterans Health Administration (VHA; 23) and Chinese Academy of Medical Sciences – Peking Union Medical College (22). Among the top 10 most productive institutions, 7 were from the United States, 2 were from France and 1 was from China. Our results indicated that the United States was still at the forefront of the world. The radar chart in Figure 4A illustrates the top 20 most productive institutions in this field.

**Table 2:** The top 10 most productive institutions related to macrophages in AAA research.

Rank	Institution	Publications	Centrality	Percentage (%)
1	Harvard University (USA)	60	0.09	6.54
2	Harvard Medical School (USA)	55	0.04	5.99
3	Brigham & Women’s Hospital (USA)	52	0.26	5.66
4	University of Kentucky (USA)	42	0.25	4.58
5	UDICE-French Research Universities (French)	33	0.14	3.59
6	Stanford University (USA)	32	0.08	3.49
7	Institut National de la Sante et de la Recherche Medicale (Inserm; French)	31	0.08	3.38
8	US Department of Veterans Affairs (USA)	24	0.17	2.61
9	Veterans Health Administration (VHA; USA)	23	0.09	2.51
10	Chinese Academy of Medical Sciences – Peking Union Medical College (China)	22	0.08	2.40

AAA = abdominal aortic aneurysm.

Additionally, we used Citespace software to create an institutional collaboration analysis. As shown in Figure 4B, each node represented an institution, and the line between nodes represented their collaboration relationship. The size of the nodes reflected their frequency. The nodes with the pick circle represented higher centrality. As for author analysis, the top 10 most published authors and cited coauthors were listed in Table 3, accounting for 22.22% of the total number of publications. Among them, Daugherty, Alan from the USA published the most publications (28), followed by Shi, Guo-ping (25), and Upchurch, Gilbert R (23). Interestingly, 7 of the top 10 most productive authors were from the USA and the remaining authors were from China (2) and Australia (1). Besides, Libby, peter had the highest average number of citations and H-index. Furthermore, we used the VOSviewer software to classify authors into different clusters. Different clusters represented cooperation among authors. As shown in Figure 4C, the minimum number of documents of an author was set as 7, a total of 51 authors met the thresholds. We could see that Daugherty, Alan worked closely with Davis, Cassis, Rateri, Howatt, Balakrishnan, Subramanian and other authors in different clusters(Shi Guoping, Libby, Thompson, Weintraub, Golledge, Moran, Dalman and Xu). Cited coauthors are defined as 2 or more authors who are simultaneously cited in 1 or more publications. The most frequently cited authors could reflect the author’s academic contribution. Among the 20713 cited coauthors, 17 authors had at least 100 citations. As shown in Figure 4D, red clusters such as Daugherty, Alan and Golledge, J were mainly involved in the molecular mechanism, experimental model and therapeutic target for AAA. They provided fresh insights into AAA experimental design and guidelines for AAA prevention.^[12–14]^ The authors from green clusters such as Thompson, RW and Newman, KM focused on the immune responses, and pathogenesis of AAA formation.^[15–17]^ The authors from bule clusters such as Longo, Gm and Newby mainly focused on the role of MMPs in AAA research.^[18,19]^ They found that deficiency of MMP-2 and MMP-9 could not result in AAA formation and prolonged administration of doxycycline was safe and reduced MMP-9 plasma level in patients with small asymptomatic AAAs. These authors all contributed to exploring the underlying mechanism and novel treatment for AAA.

**Table 3:** The top 10 authors and co-cited authors related to macrophages in AAA research.

Rank	Author	Counts	Citations	Average number of citations	H-index	Co-cited author	H-index	Citations
1	Daugherty, Alan (USA)	28	1464	52.29	71	Daugherty, Alan (USA)	71	539
2	Shi, Guo-ping (China)	25	946	37.84	58	Golledge, J (Australia)	66	366
3	Upchurch, gilbert R (USA)	23	759	33	62	Thompson, RW (USA)	49	272
4	Dalman, Ronald l. (USA)	22	1220	55.45	27	Longo, GM (USA)	16	269
5	Libby, Peter (USA)	21	1713	81.57	200	Shimizu, K (Japan)	8	186
6	Golledge, J (Australia)	19	560	29.47	66	Pyo, R (USA)	9	154
7	Sukhova, Galina K. (USA)	18	918	51	72	Curci, JA (USA)	30	141
8	Liu, Bo (USA)	17	744	43.76	33	Sakalihasan, n (Belgium)	22	141
9	Lu, Guanyi (China)	16	475	29.69	24	Xiong, WF (USA)	20	134
10	Su, Gang (China)	15	470	31.33	46	Lederle, FA (USA)	45	131

AAA = abdominal aortic aneurysm.

### 3.4. Distribution of journals and co-cited journals

**Figure 5.**
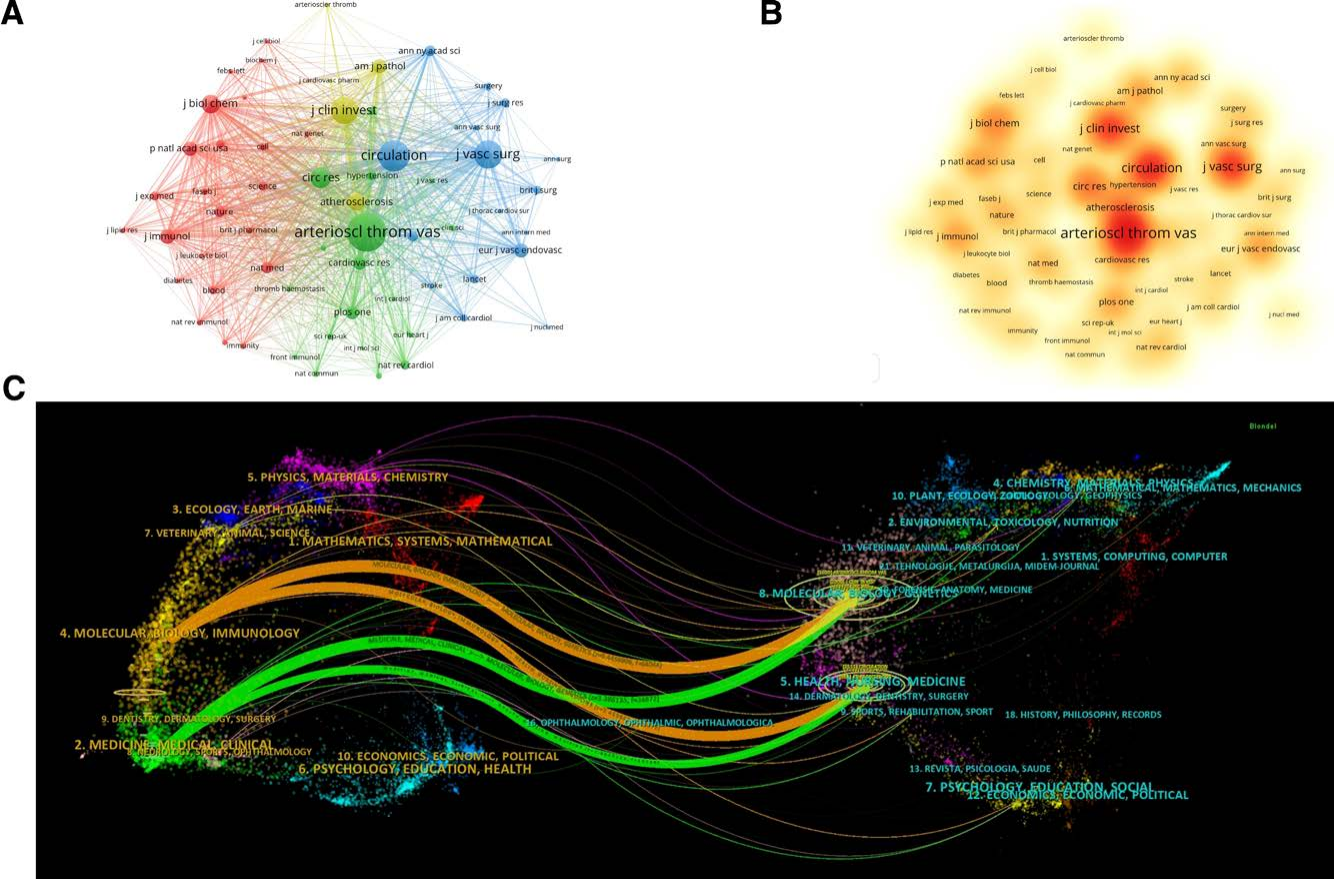
Network visualization map of journals involved in macrophages on AAA research. (A) Visualization map of academic journals generated by VOSviewer software. (B) Density visualization of academic journals. Journals with high frequencies are represented as red. The higher frequencies the journal with, the redder the journal represents. (C) The dual-map overlay of macrophage-AAA research generated by Citespace. The left side represents citing journals and the right side represents cited journals. AAA = abdominal aortic aneurysm.

Since 2000, 918 macrophage-related articles in AAA research have been published in 280 journals. We listed the top 10 most productive journals in Table 4. These journals published 373 articles, accounting for 37.36% of the total publications. Among these journals, 70% of the productive journals were classified in Q1 and the left in Q2 according to the JCR 2022 standards. The journal Arteriosclerosis Thrombosis and Vascular Biology published the largest number of publications (86, 9.37%), which had an IF of 10.51 in 2022, followed by the Journal of Vascular Surgery (58, 6.32%), Atherosclerosis (43, 4.68%), and Plos One (43, 4.68%). Circulation Research had the highest IF (23.218) in 2022. The average IF of the top 10 most productive journals was 8.67. The number of co-citations could be seen as the relevance of the literature in terms of content, so we constructed a co-citation relationship using VOSviewer software. We summarized journals that were cited >100 times. As shown in Figure 5A, the top 5 journals with the highest link strength were Arteriosclerosis Thrombosis and Vascular Biology (total link strength 144,949), followed by Circulation (109,142), Journal of Vascular Surgery (76,610), Journal of Clinical Investigation (82,216), and Circulation Research (65,009). The node size represented the number of citations. The density visualization of cited journals is shown in Figure 5B.

**Table 4:** The top 10 most productive journals.

Rank	Journal	Publications	Percentage (%)	Citations	IF (2021)	JCR
1	Arteriosclerosis Thrombosis and Vascular Biology	86	9.37	5644	10.51	Q1
2	Journal of Vascular Surgery	58	6.32	2892	4.86	Q1/Q2
3	Atherosclerosis	43	4.68	1731	6.85	Q1
4	Plos One	43	4.68	1302	3.75	Q2
5	European Journal of Vascular and Endovascular Surgery	23	2.51	1030	6.427	Q1
6	Scientific Reports	22	2.40	408	4.997	Q2
7	Circulation Research	18	1.96	1315	23.218	Q1
8	Frontiers in Cardiovascular Medicine	18	1.96	84	5.848	Q2
9	Cardiovascular Research	16	1.74	1025	14.239	Q1
10	Clinical Science	16	1.74	422	6.0	Q1

Furthermore, we performed a visual analysis of the distribution of academic journals by the dual-map overlay of the journals. The citing journals were on the left and the cited journals were on the right. As depicted in Figure 5C, the diagram shows 4 main paths in different colors. This indicates that documents published in molecular/biology/genetics are primarily cited by researchers published in molecular/biology/immunology and medicine/medical/clinical. Similarly, documents published in the fields of health, nursing, and medicine are mainly cited by researchers in the molecular biology, immunology, and medicine/medical/clinical fields.

### 3.5. Analysis of keywords

**Figure 6.**
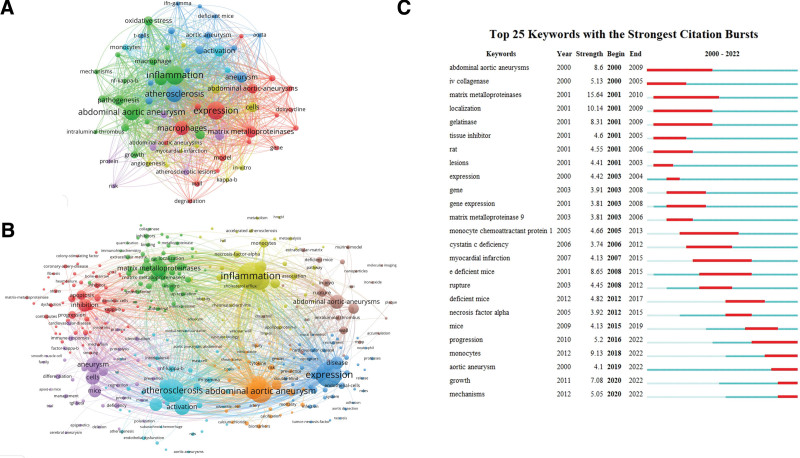
Network visualization map of keywords involved in macrophages on AAA research. (A) Network analysis of keywords appearing >20 times. Of the 3467 keywords, 67 had at least 20 co-occurrences. The node size represents the frequency of keyword occurrences. (B) Keyword cluster analysis. Different color clustering represents the respective cluster class. (C) The top 25 keywords with the strongest citation bursts. The red bar reflects the citation burst. AAA = abdominal aortic aneurysm.

The article’s keywords are considered an important index for representing research hotspots in this field. Researchers use keyword co-occurrence analysis to analyze hotspots and research trends. The keywords co-occurrence visualization map was constructed by using VOSviewer software. The top 20 most frequent keywords were listed in Table 5. As shown in Figures 6A, 67 high-frequency keywords appeared >20 times. Among these, “inflammation,” “expression,” “atherosclerosis,” “abdominal aortic aneurysms” and “macrophages” were the most frequent. To identify potential hotspots in the field of macrophages in AAA research, we performed cluster analysis of keywords that appeared more than 10 times. A total of 8 different colored clusters represented 8 potential research hotspots. As shown in Figure 6B, the largest cluster (red) consisted of 61 keywords, including inhibition, apoptosis, progression, kappa-b, dysfunction, macrophage activation, macrophage polarization, angiogenesis, angiotensin-converting enzyme etc. This cluster primarily focused on the immune mechanism behind AAA. The green cluster (52 keywords) mainly represented vascular remodeling with core keywords such as matrix metalloproteinases, matrix metalloproteinase-9, ECM localization, collagenase, elastase and vascular remodeling. The blue cluster mainly represented immune responses with 47 keywords, mainly including expression, receptor, cytokines, angiotensin ii, disease, double-blind, tumor-necrosis factor, necrosis. The yellow cluster, with 44 keywords, mainly represented the inflammatory mechanisms behind AAA with core keywords such as inflammation, necrosis-factor-alpha, monocytes, cholesterol efflux, vascular inflammation and accelerated atherosclerosis. The purple cluster mainly represented epigenetic regulation of AAA with 42 keywords including aneurysm, cells, epigenetics, DNA methylation, family, mutation, immune responses, innate immunity and autophagy. The cyan cluster with 39 keywords mainly focused on the oxidative stress and biological research including keywords such as atherosclerosis, oxidative stress, NADPH oxidase, hypoxia, reactive oxygen species, endothelial dysfunction, T cells, NF-κB and IFN-γ. The orange cluster with 38 keywords focused more on risk factors and vascular wall microenvironment, with keywords including abdominal aortic aneurysm, risk factors, mortality, calcification, calcium chloride, biomarkers, shear stress and cytokine. The brown cluster had 28 keywords, including abdominal aortic aneurysms, rupture, in vivo, murine model, deficient mice, intraluminal thrombus, atherosclerotic plaques, periarterial application, molecular imaging and nanoparticles. This cluster mainly represented experimental research more pertained to AAA-associated experimental model and clinical translation.

**Table 5:** The top 10 most frequent and centralized keywords related to macrophages in AAA research.

Rank	Keyword	Counts	Rank	Keyword	Counts
1	Inflammation	322	11	Cells	106
2	Expression	296	12	Abdominal aortic-aneurysms	101
3	Atherosclerosis	247	13	Inhibition	91
4	Abdominal aortic aneurysm	244	14	Disease	88
5	Macrophages	195	15	Oxidative stress	87
6	Smooth-muscle-cells	146	16	Mice	82
7	Matrix metalloproteinases	129	17	e-deficient mice	73
8	Activation	121	18	Abdominal aortic-aneurysm	63
9	Aneurysm	115	19	Macrophage	62
10	Pathogenesis	108	20	Angiotensin ii	59

AAA = abdominal aortic aneurysm.

**Figure 7.**
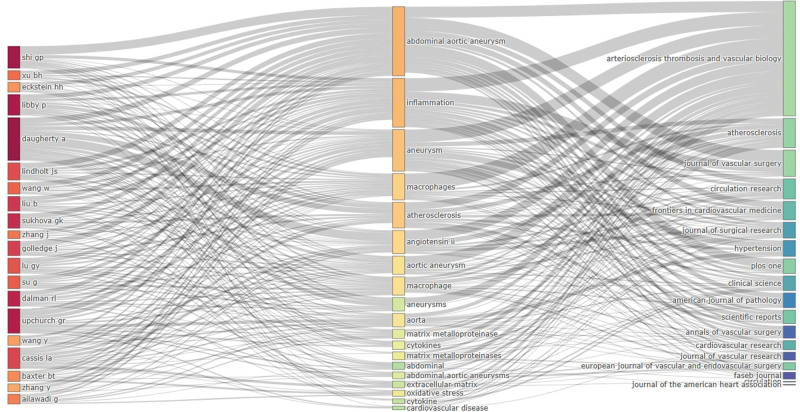
Three-field plot of authors, keywords and journals in the field of macrophage-related AAA research. AAA = abdominal aortic aneurysm.

Additionally, the top 25 keywords with the strongest citation burst were shown in Figure 6C. Matrix metalloproteinases, localization and monocytes were the top 3 keywords with the highest strength. In the early stage of macrophage-related AAA research, researchers focused more on matrix metalloproteinases and vascular remodeling. In recent years, the focus has shifted to areas such as monocytes and exploration of the mechanisms behind AAA progression. This citation burst trend has continued into 2022 and is expected to persist in the future. Furthermore, a 3-field plot analysis was performed to better illustrate the results. In Figure 7, it’s evident that Daughterty A was the most productive author. The 3-field plot further demonstrated the relationship among authors, keywords and journals in this field.

### 3.6. Analysis of references

**Figure 8.**
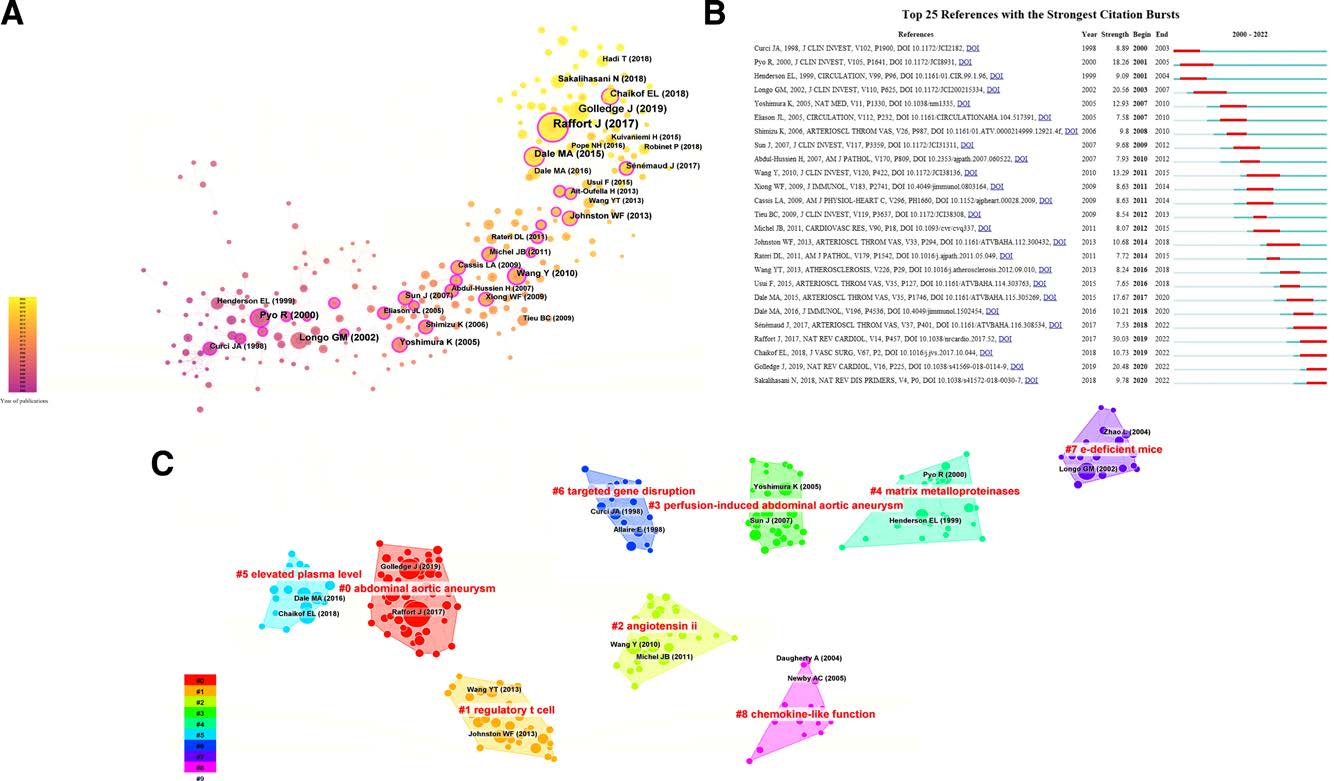
Network visualization map of references involved in macrophages on AAA research. (A) Network visualization map of cited references. (B) The top 25 references with the strongest citation bursts. (C) The clustered network map of co-cited references related to macrophages in AAA research based on Citespace. The size of the nodes in the map represents the number of co-citations, and nodes with a purple outer circle indicate significant influence in the field. AAA = abdominal aortic aneurysm.

The co-citation network referred to a network of references co-cited by 1 or more articles at the same time. The article with higher co-citation indicated it had more significant influence in this field. In the present study, the co-citation network was identified by using Citespace software. The top 10 most frequent cited references related to macrophages in AAA research were shown in Table 6. The paper published by Raffort J had the highest citations (87), followed by Golledge J (52), and Longo GM (38). Raffort J summarized the current role of monocytes and macrophage play in AAA progression [22]. The most recent article provides an update on the latest findings regarding the development of AAA and summarizes current medical treatments for AAA [23]. Figure 8A showed a visualization map of the co-citation network of cited references. The cluster analysis of co-cited references helps researchers explore current hot spots and research directions in specific fields. As shown in Figure 8B, these co-cited references were divided into 9 clusters: Abdominal aortic aneurysms (cluster #0), regulatory T cell (cluster #1), angiotensin ii (cluster #2), perfusion-induced abdominal aortic aneurysms (cluster #3), matrix metalloproteinases (cluster #4), elevated plasma level (cluster #5), targeted gene disruption (cluster #6), e-deficient mice (cluster #7), chemokine-like function (cluster #8). Additionally, the top 25 references with the strong citation burst were listed in Figure 8C. The blue line represented the time span from 2000 to 2022 and the time interval of citation burst was represented by the red line.

**Table 6:** The top 10 cited references related to macrophages in AAA research.

Rank	Title	Author	Year	Journal	Citations	DOI
1	Monocytes and macrophages in abdominal aortic aneurysm	Raffort J	2017	Nat Rev Cardiol	87	10.1038/nrcardio.2017.52
2	Abdominal aortic aneurysm: update on pathogenesis and medical treatments	Golledge J	2019	Nat Rev Cardiol	52	10.1038/s41569-018-0114-9
3	Macrophage expression of active MMP-9 induces acute plaque disruption in apoE-deficient mice	Longo GM	2002	J Clin Invest	38	10.1172/JCI200215334
4	Inflammatory cell phenotypes in AAAs: their role and potential as targets for therapy	Dale MA	2015	Arterioscl Throm Vas	37	10.1161/ATVBAHA.115.305269
5	Targeted gene disruption of matrix metalloproteinase-9 (gelatinase B) suppresses development of experimental abdominal aortic aneurysms	Pyo R	2000	J Clin Invest	34	10.1172/JCI8931
6	The Society for Vascular Surgery practice guidelines on the care of patients with an abdominal aortic aneurysm	Chaikof EL	2018	J Vasc Surg	29	10.1016/j.jvs.2017.10.044
7	TGF-beta activity protects against inflammatory aortic aneurysm progression and complications in angiotensin II-infused mice	Wang Y	2010	J Clin Invest	28	10.1172/JCI38136
8	Abdominal aortic aneurysms	Sakalihasani N	2018	Nat Rev Dis Primers	23	10.1038/s41572-018-0030-7
9	Genetic and pharmacologic disruption of interleukin-1β signaling inhibits experimental aortic aneurysm formation	Johnston WF	2013	Arterioscl Throm Vas	22	10.1161/ATVBAHA.112.300432
10	Regression of abdominal aortic aneurysm by inhibition of c-Jun N-terminal kinase	Yoshimura K	2005	Nat Med	22	10.1038/nm1335

AAA = abdominal aortic aneurysm.

There are representative articles on macrophage and AAA. The reference with the highest citation burst was published by Raffort J in 2017. In this paper, the author wrote a review describing the role of monocytes and macrophage play in AAA pathogenesis. Raffort J suggested that distinct monocyte and macrophage subsets played a crucial role in all stages of AAA development, from initial development to rupture. The article “Abdominal aortic aneurysm: update on pathogenesis and medical treatments” was published in Nat Rev Cardiol by Golledge J in 2017. In this review, Golledge et al summarized the latest research findings on AAA pathogenesis. Additionally, this review updated potential medical therapies for AAA including medical interventions for small AAAs. It’s worth noting that that most of the early references focused on the inflammatory responses, clinical treatment and targeted therapy in AAA. The article “Macrophage expression of active MMP-9 induces acute plaque disruption in apoE-deficient mice” was published in J Clin Invest in 2002. In this study, Longo GM and his colleagues found that expression of an autoactivating form of MMP-9 in macrophages greatly aggravated elastin degradation and contributed to significant plague disruption in apoE^−/−^ mice. His study suggested that MMP9 should be considered a potential therapeutic target for stabilizing rupture-prone plaques. In 2000, Robert W. Thompson published a study titled “Targeted gene disruption of matrix metalloproteinase-9 (gelatinase B) suppresses development of experimental abdominal aortic aneurysms.” In this study, Thompson and his team used an elastase-induced AAA model to assess the role of MMPs in AAA progression. The study suggested that increased MMPs contributed to the destruction of the aortic wall, and that treatment with a nonselective MMP inhibitor targeting MMP9 significantly slowed elastase-induced aneurysmal degeneration. Representative articles on macrophage and AAA have made significant strides in understanding the pathogenesis and treatment of AAA.

## 4. Discussion

### 4.1. General information

AAA has become the thirteenth leading cause of death in the USA, resulting in 9000 deaths each year.^[20]^ Accumulating studies suggested AAA was characterized by inflammatory cell infiltration, VSMCs dysfunction and ECM degradation.^[21]^ Thus, improving our understanding of AAA is particularly crucial. Macrophages in AAA research have received significant attention in recent years.^[22]^ Chronic macrophage-associated inflammation is a key mediator during AAA progression. This finding underscores the importance of understanding macrophage dynamics in AAA.^[23]^ This study used bibliometrics to analyze trends in macrophage-related research in AAA.

Research in macrophages in the field of AAA research was flourishing. A total of 918 English-language publications from 2000 to 2022 by 1091 institutions in 34 countries were retrieved from the WoSCC database on October 17, 2023. Our results showed a rise in publications and citations on macrophages in AAA from 2000 to 2022. A total of 918 English-language publications were retrieved, representing a more than 9-fold increase in the last 20 years, rising from 7 publications in 2000 to 73 in 2022. Surprisingly, macrophage-related AAA publications in the past 5 years (320) made up 34.9% of all articles on the topic. This suggests this field gained substantial attention in recent years.

The number of publications was a key measure of a country, institution, and author’s contributions to this field. The United States, China and Japan were the top 3 representative countries. They made up over 80% of the total publications in this field. The United States was still the leading country in this field. It had 377 publications in the past decades. Of the top 10 most productive institutions, 7 were in the US. The most influential institution in macrophage-related AAA research was Harvard University. It had 60 publications, followed by Harvard Medical School (55), Brigham & Women’s Hospital (52), University of Kentucky (42), UDICE-French Research Universities (33).

Of the top 10 most productive scholars, 6 were from the US. In this domain, Daugherty, Alan (28), Shi, Guo-ping (25) and Upchurch, Gilbert R (23) ranked in the top 3. Professor Daugherty’s team concentrated on the pathogenesis of AAA and the development of protective methods for its prevention. Daugherty and his colleagues reviewed animal models, sex differences, and inflammation-related mechanisms. They also examined a potential new pathway: sclerostin, which is related to bone homeostasis. Lastly, they looked at recent therapies for AAA.^[24]^ While professor Shi, Guo-ping focused on the innate immune cell, such as eosinophils, regulator T cell and mast cell. In 2015, Shi explored the role of Tregs in an Ang-II induced AAA model and suggested that Tregs protect against AAA in humans and in experiments.^[25]^ In his recent research, Shi suggested that eosinophils effectively prevented AAA formation by regulating macrophage polarization and inhibiting the activation of NF-κB signaling pathway.^[26]^ Professor Upchurch, Gilbert R from University of Florida was the representative author who mainly focused on the clinical research of AAA. Upchurch provided valuable insights and recommendations for clinical AAA management.^[27–29]^ Libby et al^[30]^ found much in the vascular microenvironment of atrial disease, like vascular remodeling and immune balance. Notably, Libby, Peter had the highest H-index and citations. His work had a major influence in this field.

The journal analysis helped researchers find core journals in the field. It also helped them identify suitable journals for data collection and manuscript submission. Our results showed that most of these journals were in the categories of peripheral vascular disease, cardiac and cardiovascular systems, and hematology. Arteriosclerosis Thrombosis and Vascular Biology, Journal of Vascular Surgery, and Atherosclerosis were the top 3 most productive journals, suggesting these journals were interested in works regarding macrophages on AAA research. Of the top 10 most influential journals, 7 were in the JCR1 region. The top 3 were Circulation Research, Cardiovascular Research, Arteriosclerosis, Thrombosis, and Vascular Biology. They had the highest impact factors (IF > 10).

Arteriosclerosis Thrombosis and Vascular Biology are more interested in researching aneurysm pathogenesis and assessing strategies to prevent aneurysm expansion. Journal of Vascular Surgery also published high-quality clinical and laboratory articles on macrophage in AAA research. This journal is dedicated to improving the management of AAA patients and testing new hypotheses, such as the experimental murine model, in AAA research. Atherosclerosis was founded in 1970 and is mainly concentrated in the fields of pathogenesis, genetic and epigenetic basis and treatment of AAA. These journals provided groundbreaking research and new insights. These data will assist scientists in finding journals for submitting manuscripts related to macrophages and AAA.

### 4.2. Current hotspots and frontiers in macrophage-associated AAA research

The keyword co-occurrence analysis enabled researchers to understand the key aspects and evolution of macrophage research in AAA. Typical cluster analysis helped researchers find hotspots in a field. The research hotspots and frontiers in macrophage and AAA were identified as follows:

#### 4.2.1. Research in animal models of AAA

Experimental animal models offer new opportunities to investigate AAA mechanisms and identify therapeutic targets. Many experimental AAA models have been discussed across a variety of species (pigs, sheep, dogs, and rodents). However, ethical concerns and the higher costs of using larger animals remain challenges in AAA studies.^[31]^

AAA formation was considered as a complex process and 3 mouse models have been used to study the pathological conditions of AAA: transient perfusion of elastase model, periaortic application with Cacl_2_ model and subcutaneous infusion of angiotensin II model.^[19,32–34]^ These AAA-inducing models mainly depended on the mechanical injury to the arterial wall.^[35]^ Different aortic pathologies were observed in these models. To increase the validity of the findings, more and more researchers used at least 2 of the models in their study. Liu et al^[36]^ verified that TSP1 caused vascular inflammation in 3 established AAA animal model. Yue et al^[37]^ combined subcutaneous Ang II infusion with elastase perfusion to develop a new AAA rupture model. However, due to the complex mechanism of AAA development, these well-established could not fully recapitulate human AAA’s pathogenesis.

Researchers have made several modifications to address this problem. In a recent study, Lin reported that periarterial incubation with papain (an enzyme extracted from papaya latex) successfully induced AAA formation in C57BL/6J mice. This AAA model had lower mortality and was cost-effective.^[38]^ To the best of our knowledge, AngII model was the most commonly used model to investigate miRs in AAA. A review by Raffort J revealed that micro-RNAs (miRs) have emerged as key regulators of gene expression. They play an important role in vascular biology and cardiovascular disease.^[35]^ Raffort’s review summarized differential miRs expression in human and animal studies. It discussed miRs’ role in AAA’s pathogenesis and suggested that circulating miRs might be biomarkers for AAA development. Aldosterone was seen as a separate risk factor for heart disease. High aldosterone levels were linked to hypertension and heart failure.^[39]^ A recent study explored how aldosterone’s activation of mineralocorticoid receptors causes AAA. It found that deoxycorticosterone acetate plus a high-salt diet caused severe aneurysms. Treating with mineralocorticoid receptor antagonists reduced AAA formation.^[40]^ These findings provided fresh insights for the prevention of AAA. We must accurately grasp the AAA features seen in humans, thus more research on animal studies is needed.

#### 4.2.2. Inflammatory microenvironment of AAA

AAA is a complex disease with a multifactorial pathogenesis. Key contributing factors include inflammatory cell infiltration, ECM degradation, VSMC apoptosis, biomechanical vessel wall stress, and intraluminal thrombus.^[41]^ Recent studies have emphasized the role of the inflammatory microenvironment in AAA’s pathogenesis.^[42]^ Under inflammatory conditions, the vascular damage initiated.^[43]^

Recent studies suggested 2 origins of macrophages contributed to the pathogenesis of AAA: tissue-resident macrophages and circulating monocyte differentiated macrophages. The circulating monocytes are the major type of macrophages in the aortic wall. Under inflammatory conditions, circulating monocytes accumulate in aortic tissues and differentiate into macrophages. Different inflammatory stimuli cause macrophages to differentiate into distinct phenotypes (M1 and M2).^[44]^ Macrophages with pro-inflammatory effects are classified as M1 macrophage whereas M2 macrophage are with anti-inflammatory properties. And these 2 phenotypes of macrophages serve opposite roles in AAA.

M1 macrophage preferentially locate in the tunica adventitia of aorta wall and can be activated by LPS and IFN-γ. They release pro-inflammatory cytokines, like TNF-α, IL-1β, and IL-6, and nitric oxide (NO). These cytokines cause vascular inflammation and dilate the aortic wall. Conversely, M2 macrophage produce anti-inflammatory cytokines, such as TGF-β and IL-10 regulating angiogenesis and collagen deposition.^[45]^

Abnormally high ratio of M1/M2 contribute to AAA progression. AAA has been found associated with increased M1 macrophage polarization. Dale et al^[46]^ proved that M1 macrophages strongly exacerbated AAA formation in the Cacl_2_-induced AAA model. Therapeutics that target M1 polarization have proven effective in preventing AAA progression. A recent study suggested that circCdyl promoted AAA formation by inducing M1 macrophage polarization.^[47]^ Furthermore, topiramate decreased M1 macrophage activity and slowed AAA progression.^[48]^

MMPs belong a family of zinc-dependent, multidomain endopeptidases and can degrade many ECM components.^[49]^ Different cell types, including macrophages, VSMCs, endothelial cells, and neutrophils, produce MMPs. Evidence showed that MMP activation greatly affected AAA progression. MMP2 and MMP9 are gelatinases and are capable of degrading elastin and collagens in the ECM. The deletion of MMP9 significantly reduced the formation of AAA induced by Cacl2 and elastase. It’s worth noting that the role of MMP12 play in AAA is emerging.^[50]^ MMP12 is highly expressed in AAA tissues. Also, MMP2 deficiency promoted AAA development and rupture.^[51]^ Endogenous tissue inhibitors of metalloproteinases (TIMPs) were the specific endogenous inhibitor of MMPs. The imbalance between MMPs and TIMPs is considered as a crucial part of ECM degradation in AAA tissues.^[31]^ Hu et al’s^[52]^ study found that loss of TIMP3 aggravated vascular inflammation in an experimental model of AAA. These findings provide therapeutic potential for suppressing AAA development.

#### 4.2.3. Immune mechanism of AAA

The role of innate immune cells play in the pathogenesis of AAA is emerging. Solid evidence has been accumulated for the importance of the immune responses in the initiation and progression of AAA.^[53]^ Studies have shown that macrophages are the first immune cells accumulating in the aorta tissues. Macrophages can secrete cytokines. The activation of macrophage causes chronic inflammation and further destroys the stability of the aortic wall.

Single-cell sequencing analysis found that the activation of T follicular helper (Tfh) cells and macrophage polarization may cause AAA.^[54]^ In this study, the author performed subcluster analysis of myeloid cells and found 2 macrophage clusters. Macrophage cluster#1 showed upregulation of proinflammatory M1-like genes (TNF, IL-1β) and several inflammatory cytokines (CCL3L, CXCL3). In contrast, macrophage cluster#2 showed upregulation of interferon-related genes (IFI44L, IFIT3, IFITM3). The GO enrichment analysis showed that macrophage cluster#1 had high inflammatory pathway activity. In contrast, macrophage cluster#2 had high activity in antigen processing and type I interferon signaling pathways.

The role of macrophage-derived cytokines has been well-studied in various studies. Recently, a unique subset marked by Netrin 1 (Ntn1) was identified among CD11b + CD68 + Adgre1 + macrophages. Highly expressed pro-inflammatory and pro-angiogenic markers were found in Ntn1-positive macrophages. Studies also have shown that Ntn1-dificiency in macrophages significantly limited AAA progression.^[55]^

Exosomes have gained much attention in recent years due to their capability to mediate cell communication.^[56]^ Exosomes can be detected in the adventitia of AAA tissues and macrophage-derived exosomes upregulate the expression of MMP2. Intraperitoneal injection of GW4869 (an inhibitor of exosome biogenesis) significantly prevented AAA progression in CaPO_4_-induced AAA mice. The nucleotide-binding oligomerization domain-like receptor family protein 3 (NLRP3) inflammasome is a well-studied intracellular complex. It can trigger caspase-1, facilitate secretion of IL-1β and IL-18 initiating the inflammatory cascade under pathological conditions.^[57]^ Aberrant activation of NLRP3 inflammasome and pyroptosis is involved in the pathogenesis of AAA. The purinergic receptor (P2X7, an important inflammatory mediator) was mainly confined in macrophage in AAA specimen. Previous studies have shown that P2X7-difiency limited AAA formation. In contrast, P2X7-agonists promoted AAA development.^[5]^ Also, miR-17-5p from ADSC-exos protected the integrity of vascular wall. It prevented AAA progression by targeting the TXNIP-NLRP3 inflammasome signaling pathway. These data highlight the central pathogenic role of macrophage-related inflammatory microenvironment in AAA progression.^[58]^

#### 4.2.4. Clinical translation and molecular imaging

Molecular imaging target core players in the pathogenesis of AAA. It can provide fresh insights and novel diagnostic approach for AAA visualization. This helps in making personalized treatment decisions. The current management of AAA relies on abdominal ultrasound or CT to assess the morphological features of aneurysms. However, these anatomy-based diagnostic approaches fail to capture the risk of AAA’s rupture. Through characterization and quantification of biological processes involved in AAA development, molecular imaging can effectively improve the risk stratification of AAA. It’s urgent to search for imaging biomarkers which can identify the risk of rupture independent of the diameter of AAA. Previous findings showed that macrophage-driven inflammation promotes AAA progression. Therefore, noninvasive imaging of macrophage buildup in lesions may help researchers find rupture-prone lesions among the stable ones.

A study published in the journal Theranostics suggested that phagocytic uptake of CT contrast nanoparticles could be leveraged to detect vascular inflammation in preclinical AAA models by CT.^[59]^ In this study, Sadeghi et al tested several nanoparticle CT agents in macrophage cell lines. Exitron showed unique uptake in macrophages. Furthermore, his team found that Exitron specific accumulated within macrophages and created CT signal that correlated with CD68 expression. His team also suggested Exitron signal had predictive value of AAA outcome.

Imaging the vascular wall’s inflammation may help researchers better evaluate AAA states and formulate effective targeted treatment plan. The ultrasmall superparamagnetic particles of iron oxide (USPIOs) have been used as alternative MR contrast agents in cardiovascular field. USPIOs are gradually engulfed by macrophages and accumulate at inflammatory sites. Their specific activities have made them indicators of macrophage activity in the cardiovascular field. A study by Newby found that uptake of USPIOs in AAA was closely corelated with aneurysms growth rate and could be used to identify cellular inflammation in AAAs.^[60]^ Molecular imaging brings many benefits. But, more preclinical tests in animal models remain to be done.

### 4.3. Limitations

This study presents the first bibliometric analysis of macrophages in AAA research, focusing on knowledge maps, research hotspots, and future trends. There are still some limitations in our study. Firstly, only English articles and reviews from Sci-Expanded-Indexed database were included in our study, and this limitation may lead to the exclusion of significant research findings. Secondly, our study based on articles published between 2000 and 2022, meaning our study was relevant to this research period, newly published studies were not included in the final bibliometric pool after 2023. We hope our future studies will focus on broader databases and we want to learn how global research studies macrophages in AAA.

## 5. Conclusion

This study presents the first systematic bibliometric and visualization analysis of macrophages in AAA research. We analyzed the global trends, regional distribution, collaboration among authors and institutions. In addition to trends and regional distribution, we also analyzed influential journals and identified potential future directions for research. Our results suggested that research on macrophage in AAA field has gained wide attention. The exploration of established animal models, the inflammatory microenvironment related to macrophages, the immune mechanism related to macrophages, clinical translation and molecular imaging research remained future research directions in this field. Our findings shed light on current research hotspots and offer valuable guidance for future studies on macrophages in AAA.

## Acknowledgments

The authors would like to thank the Key Laboratory of Pathogenesis, Prevention and Therapeutics of Aortic Aneurysms at the First Hospital of China Medical University. We thanked for their valuable suggestions for data analysis.

## Author contributions

**Conceptualization:** Dongyu Li, Shiyang Bao.

**Data curation:** Liang Zhang, Dongyu Li.

**Formal analysis:** Liang Zhang, Shiyang Bao.

**Investigation:** Liang Zhang.

**Software:** Liang Zhang, Dongyu Li, Shiyang Bao.

**Supervision:** Liang Zhang, Shiyang Bao.

**Validation:** Liang Zhang, Dongyu Li.

**Visualization:** Liang Zhang, Dongyu Li, Shiyang Bao.

**Writing – original draft:** Shiyang Bao.

**Writing – review & editing:** Dongyu Li.
